# Cannabis as a substitute for alcohol and other drugs

**DOI:** 10.1186/1477-7517-6-35

**Published:** 2009-12-03

**Authors:** Amanda Reiman

**Affiliations:** 1 School of Social Welfare, University of California, Berkeley, 120 Haviland Hall, Berkeley, CA 94720, USA

## Abstract

**Background:**

Substitution can be operationalized as the conscious choice to use one drug (legal or illicit) instead of, or in conjunction with, another due to issues such as: perceived safety; level of addiction potential; effectiveness in relieving symptoms; access and level of acceptance. This practice of substitution has been observed among individuals using cannabis for medical purposes. This study examined drug and alcohol use, and the occurrence of substitution among medical cannabis patients.

**Methods:**

Anonymous survey data were collected at the Berkeley Patient's Group (BPG), a medical cannabis dispensary in Berkeley, CA. (N = 350) The sample was 68% male, 54% single, 66% White, mean age was 39; 74% have health insurance (including MediCal), 41% work full time, 81% have completed at least some college, 55% make less than $40,000 a year. Seventy one percent report having a chronic medical condition, 52% use cannabis for a pain related condition, 75% use cannabis for a mental health issue.

**Results:**

Fifty three percent of the sample currently drinks alcohol, 2.6 was the average number of drinking days per week, 2.9 was the average number of drinks on a drinking occasion. One quarter currently uses tobacco, 9.5 is the average number of cigarettes smoked daily. Eleven percent have used a non-prescribed, non OTC drug in the past 30 days with cocaine, MDMA and Vicodin reported most frequently. Twenty five percent reported growing up in an abusive or addictive household. Sixteen percent reported previous alcohol and/or drug treatment, and 2% are currently in a 12-step or other recovery program. Forty percent have used cannabis as a substitute for alcohol, 26% as a substitute for illicit drugs and 66% as a substitute for prescription drugs. The most common reasons given for substituting were: less adverse side effects (65%), better symptom management (57%), and less withdrawal potential (34%) with cannabis.

**Conclusion:**

The substitution of one psychoactive substance for another with the goal of reducing negative outcomes can be included within the framework of harm reduction. Medical cannabis patients have been engaging in substitution by using cannabis as an alternative to alcohol, prescription and illicit drugs.

## Background

It has been observed that those who use large amounts of cannabis frequently use other drugs as well, especially alcohol. This can create a potential synergistic effect, resulting in increased harms [1-4]. Economic research has looked at the substitution and complimentarity of particular substances by modelling the effects of price fluctuation on use, although the limits of such research have been noted [5]. When considering youth, Pacula has found cannabis and alcohol to be compliments. As beer prices rose, cannabis use declined [6]. This could potentially be because the introduction of alcohol into an adolescent environment increases the likelihood of other substance being brought into that environment; once the presence of alcohol decreases, the presence of other substances might decrease as well. Among adults, amphetamine has been found to be a substitute for those who's drug of choice is alcohol, and alcohol as a substitute for those who cannot obtain MDMA and cocaine [7,8]. This research suggests that through various patterns, individuals are making personal decisions about alcohol and drug substitution.

For the purposes of this study, substitution was operationalized as the conscious choice to use one drug (legal or illicit) instead of, or in conjunction with, another due to issues such as: perceived safety; level of addiction potential; effectiveness in relieving symptoms; access and level of acceptance. The substitution of cannabis for alcohol and other drugs has been observed among individuals using cannabis for medical purposes. Medical cannabis patients are regular cannabis users with a stable supply, and their access to cannabis not granted under a standardized prescription system, yet still legitimized by a doctor's recommendation (self-medication). This, in addition to the legal protection given to patients in California, increases the freedom of choice regarding the use of cannabis as a substitute among this population. A survey of 11 medical cannabis doctors in California found that all doctors had seen patients who were using cannabis as a substitute for alcohol. Furthermore, one said that over half of her patients reported preferring cannabis to alcohol, and another reported that 90% of his patients reduced their alcohol use after beginning the use of medical cannabis [4]. The dual use of alcohol and cannabis has been observed in several research studies on medical cannabis patients. First, previous alcohol abuse was reported in 59 of 100 medical cannabis users in a University of California, San Francisco study. Furthermore, 16 of 100 subjects reported previous alcohol dependence [9].

Beyond the population of medical cannabis patients, substituting cannabis or other drugs for alcohol has been described as a radical alcohol treatment protocol. If alcohol negatively affects a person's level of functioning, cannabis or another drug might be an alternative for the user. Charlton has suggested that the radical approach of substitution with substances such as benzodiazepine might be used to address heavy alcohol use in the British Isles by incorporating the idea of self-medication into his discussion by his assertion that "the drug-substitution strategy is based on the assumption that most people use lifestyle (recreational) drugs rationally for self-medication purposes" (p. 457). It is posited that people might substitute a safer drug with less negative side-effects if it were socially acceptable and available [10].

The first cannabis substitution study was a single subject study conducted by Tod Mikuriya in 1970, in which a female (age 49) who was an alcoholic was instructed to substitute cannabis for alcohol. The subject was also administered Antabuse to assist in her abstention from alcohol. The subject reported increased ego strength, useful behaviour, ability to control cannabis intake, euphoria and tranquilization. In addition, there were improvements in concentration, disposition, physical health, ability to revisit social situations and ability to appropriately express anger [11]. The issue was revisited in 2001 with a study of 104 medical cannabis patients in California who used cannabis in an effort to stop the use of other drugs, in particular alcohol. For example, participants may have been previous alcoholics who have replaced their alcohol use with a daily regimen of cannabis. Demographic data were collected as well as information on family alcohol history and alcohol and cannabis usage patterns. The authors included both descriptive statistics and excerpts from interviews. With respect to family alcohol history, 55% of participants reported having one or two alcoholic parents. Most of the participants (90%) listed alcohol as their primary drug of choice, although a few participants had also had addiction issues with heroin, cocaine, amphetamine and other drugs. One interesting finding in this study is that 45% of patients reported using cannabis to relieve pain that they suffered as a result of an alcohol related injury [12].

Cannabis substitution has also been discussed as part of a harm reduction framework. A record review of 92 medical cannabis patients who used marijuana as a substitute for alcohol was conducted with the goal of describing these patients and determining the reported efficacy of treatment. Fifty-three percent of participants reported being raised by at least one alcoholic/addict parent. Concerning reported health problems, 64% of the sample identified alcoholism or cirrhosis of the liver as their presenting problem. Thirty six percent identified themselves as alcohol abusers but listed another health problem as their primary concern. As in Mikuriya's 2001 study, 21% of the sample reported having been injured in an alcohol related incident. When addressing the efficacy of cannabis as a substitute for alcohol, all participants reported cannabis substitution as very effective (50%) or effective (50%). Ten percent of the patients reported being abstinent from alcohol for more than a year and attributed their success to cannabis. Twenty one percent of patients had a return of alcoholic symptoms when they stopped using cannabis. Reasons for stopping the cannabis use ranged from entering the armed forces to being arrested for using cannabis [13].

Previous alcohol use, treatment, and substitution were also documented in a sample of 130 medical cannabis patients in the San Francisco Bay Area. Twenty four had reported previous alcohol treatment. Half of the sample reported using cannabis as a substitute for alcohol, 47% for illicit drugs and 74% using it as a substitute for prescription drugs. The most common reason reported for using cannabis as a substitute was fewer side effects from cannabis and better symptom management from cannabis [14].

The personal health practice of substitution among medical cannabis patients can provide information concerning non-traditional and alternative means used by individuals to personally address their health issues without official involvement in the health care system. Furthermore, examining substitution among this population might translate into the development of more effective, client-centred treatment practices within the field of addiction.

## Methods

The survey sample for this study consisted of 350 medical cannabis patients between the ages of 18 and 81 from the San Francisco Bay Area, California. Participants are members of Berkeley Patients Group (BPG), a medical cannabis dispensing collective in Berkeley, CA. The sample was 68.4% male (N = 238), 66.2% White (N = 231) and 14.6% Multi-racial (N = 51). The mean age was 39.43.

A survey was created by the researcher, with portions adapted from a patient survey administered by Dr. Frank Lucido at his medical practice in Berkeley, CA. The survey had five sections: demographic information, medical information, cannabis use pattern, alcohol and drug use and service utilization. Participants were asked the quantity and frequency of alcohol, tobacco and drug (prescription and illicit) use as well as current and past alcohol and/or drug treatment. Participants were also asked about whether they use cannabis as a substitute for alcohol, illicit drugs or prescription drugs and why to investigate medical cannabis as a treatment for alcohol and/or drug dependence.

The survey data were collected by the researcher at BPG. The researcher approached patients as they came into BPG and asked if they would like to participate in an anonymous survey being conducted by BPG. If patients were not able to fill out the survey, it was administered by the researcher. The survey included an explanation of the study and the right to refuse to participate or to stop the survey at any time. Data collection occurred for the most part during the hours of 1-5 pm and took place during the week and on weekends. Data were analyzed in SPSS, and frequencies were calculated.

There are several limitations of this study. First, due to the close proximity to the campus of the University of California, Berkeley, there might be an over-representation of college students in this sample. This might affect data on employment status, age, marital status, income and to a lesser extent, gender and race. Secondly, although data were collected in the middle of the day regularly for several months, it is possible that some patients might come to BPG at times when data collection was not occurring. Furthermore, patients who are extremely ill might not be able to stay and fill out a survey. The sample itself prevents the generalization of these results to the greater population of cannabis users, as medical cannabis patients might differ in substantial ways from the general population, especially concerning areas of substance using behaviour, and patients from Berkeley Patient's Group may not represent the greater population of medical cannabis patients. Furthermore, there are not formal measures of alcohol drug related problems on the survey, making it impossible to explore the behavioural implications of cannabis substitution. Finally, although the survey was anonymous, the legal status of medical cannabis might prevent some patients from filling out surveys and some participants from being completely forthcoming with information. Furthermore, although the practice of substitution was described to participants in the survey, the data do rely on self report and the participant's own reality concerning their substitution behaviour.

## Results

### Alcohol, Tobacco and Other Drug Use

Fifty three percent of the sample reported that they currently drink alcohol. The average number of drinking days per week was 2.63 (N = 180). The average number of drinks on drinking days was 2.88 (N = 163). One quarter of the sample currently smoke tobacco. The average number of cigarettes smoked per day is 9.54 (N = 80). Eleven percent of the sample reported using a drug other than cannabis, a prescription or over the counter drug in the past 30 days. Cocaine, MDMA and Vicodin were reported most frequently (N = 5), followed by LSD (N = 4), mushrooms and Xanax (N = 3).

### Treatment

One quarter of the sample reported growing up in an alcoholic or abusive household, 16.4% reported previous alcohol or substance abuse treatment, and 2.4% are currently in a 12-step or some other type of substance abuse or alcohol dependence program.

### Substitution

As shown in Table 1, forty percent of the sample reported using cannabis as a substitute for alcohol, 26% reported using it as a substitute for illicit drugs, and 65.8% use it as a substitute for prescription drugs. Referring to Table 2, sixty five percent reported using cannabis as a substitute because it has less adverse side effects than alcohol, illicit or prescription drugs, 34% use it as a substitute because it has less withdrawal potential, 17.8% use it as a substitute because its easier to obtain cannabis than alcohol, illicit or prescription drugs, 11.9% use it as a substitute because cannabis has greater social acceptance, 57.4% use it as a substitute because cannabis provides better symptom management, and 12.2% use it as a substitute for some other reason.

**Table 1:** Percent of sample reporting using cannabis as a substitute

	N	%
Alcohol substitute	134	40
Illicit drug substitute	87	26
Prescription drug substitute	219	65.8

**Table 2:** Reasons for using cannabis as a substitute

	N	%
Less adverse side effects	197	65
Less withdrawal potential	103	34
Ability to obtain cannabis	54	17.8
Greater social acceptance	36	11.9
Better symptom management	174	57.4
Other reason	37	12.2

## Discussion

Research has suggested that medical cannabis patients might use more alcohol than non patients, and might have a higher instance of alcohol abuse than the general population [3,9]. Drinking patterns among the BPG sample were average, with 53.4% of the sample being current drinkers, the mean number of drinking days per week being 2.63 and the mean number of drinks on occasion being 2.88. When looking at the national rate of alcohol use, 55% of the U.S. population 18+ is a current drinker, compared to 53% of the BPG sample. The national data report 7.8% of the 18+ national sample have used an illicit drug in the past month, compared to 11% of the BPG sample [15]. The study of 100 patients from San Francisco found a much higher rate of tobacco smoking (78% vs. 24.9% of the BPG sample) [9].

When considering previous alcohol and/or substance abuse treatment, 16.4% of the BPG sample reported previous treatment for alcohol or substance abuse; this was the same percentage found in Reiman's sample of 130 medical cannabis patients [14]. Mikuriya found in 2001 and 2004 that 55% and 53% of patients respectively reported having one or two alcoholic parents [12,13]. One quarter of this sample reported growing up in an alcoholic or abusive household.

As previously discussed, research on medical cannabis patients has alluded to the use of cannabis as a substitute for alcohol, illicit or prescription drugs [9-13]. This phenomenon was also reflected in the data on substitution from the BPG sample, as 40% of participants reported using cannabis as a substitute for alcohol, 26% as a substitute for illicit drugs and 65.8% as a substitute for prescription drugs. These substitution rates were very similar to those found by Reiman [14]. Additionally, three patients noted during the survey that they used cannabis to quit smoking tobacco.

Eighty five percent of the BPG sample reported that cannabis has much less adverse side effects than their prescription medications. Additionally, the top two reasons listed by participants as reasons for substituting cannabis for one of the substances previously mentioned were less adverse side effects from cannabis (65%) and better symptom management from cannabis (57.4%).

## Conclusion

The substitution of one psychoactive substance for another with the goal of reducing negative outcomes can be included within the framework of harm reduction. Medical cannabis patients have been engaging in substitution by using cannabis as an alternative to alcohol, prescription and illicit drugs. This brings up two important points. First, self determination, the right of an individual to decide which treatment or substance is most effective and least harmful for them. If an individual finds less harm in cannabis than in the drug prescribed by their doctor, do they have a right to choose? Secondly, the recognition that substitution might be a viable alternative to abstinence for those who are not able, or do not wish to stop using psychoactive substances completely. Due to a potential conflict between the use of medical cannabis and philosophies of recovery programs such as Alcoholics Anonymous, some dispensaries offer harm reduction based recovery groups aimed at those in recovery who use medical cannabis. Mikuriya has suggested the development of 12 Step groups tailored towards those who want to take advantage of the cost free, fellowship driven nature of 12 Step programs, but wish to use cannabis actively during recovery [13]. The lack of drug and alcohol related problem measures utilized in this study calls for a further investigation into the relationship of such problems and the use of cannabis as a substitute. To that end, more research needs to be done on the possibilities for substitution that lie in the field of addiction, and on the individuals who have already successfully incorporated substitution into their health care regime.

## Competing interests

The author declares that she has no competing interests.

## Author information

Amanda Reiman MSW, PhD, is currently the Coordinator of Academic Programs and a Lecturer in the School of Social Welfare at the University of California, Berkeley. She is also the current Chairwoman of the Berkeley Medical Cannabis Commission.

## Authors' contributions

AR conceived the study design, created and administered the survey, entered the data into the computer, analyzed the data and wrote the final report.
